# Small cell carcinoma of the bladder with coexisting prostate adenocarcinoma: two cases report and literature review

**DOI:** 10.1186/s12894-020-00705-3

**Published:** 2020-08-28

**Authors:** Yanxia Liu, Hongming Xu, Bin Wu, Shuguang Liu, Qiang Luo

**Affiliations:** 1 Department of Pathology, Jiangyin People’s Hospital, Affiliated Jiangyin Hospital of the Southeast University Medical College, Jiangyin, 214400 Jiangsu Province China; 2 Department of Urology, Jiangyin People’s Hospital, Affiliated Jiangyin Hospital of the Southeast University Medical College, Jiangyin, 214400 Jiangsu Province China; 3 Department of Urology, Tianjin Jinnan Hospital, Tianjin, 300350 China

**Keywords:** Small cell carcinoma, Urinary bladder, Prostate, Adenocarcinoma

## Abstract

**Background:**

Primary small cell carcinoma of the bladder (SCCB) is a rare disease of the genitourinary tract and reported limitedly. SCCB is very aggressive and always mixed with other histologic components, but coexistence of SCCB and prostate adenocarcinoma is extremely rare.

**Cases presentation:**

Two aged males (72 and 58 years) were included in this study. Both of them presented with gross hematuria as initial symptom. Magnetic resonance imaging (MRI) demonstrated protruding lesions in the urinary bladder. Pathological examination after radical cystectomy and prostatectomy showed the concurrence of SCCB and prostate adenocarcinoma. One patient died of liver and lung metastasis 8 months after surgery, and the other patient was still alive after 19 months of follow-up.

**Conclusion:**

In this paper, we reported two unusual cases of coexistence of SCCB and prostate adenocarcinoma, and reviewed relative literatures with respect to the epidemiology, clinical features, pathologic features, diagnosis, treatment and prognosis of SCCB.

## Background

Small cell carcinoma occurs mainly in the lung, which occupies about 15–20% pulmonary malignant tumors. Besides, this disease could also exist in extra pulmonary sites, such as skin, gastrointestinal and urinary tract [1, 2]. Small cell carcinoma of the bladder (SCCB) accounts for 0.35–0.7% of bladder tumors and indicates aggressive biological behavior and poor prognosis [3]. SCCB is usually admixed with other pathological subtypes, like urothelial carcinoma and squamous cell carcinoma [4]. Prostate cancer is the most commonly diagnosed cancer and the second leading cause of cancer related death in men around the world. Nevertheless, coexistence of SCCB and prostate adenocarcinoma in one patient was extremely scarce, which brought certain challenges to the treatment and prognosis of patients [5, 6]. Here, we described two cases of such rare malignancy who underwent surgery in our institution.

## Cases presentation

### Case 1 #

**Fig. 1:**
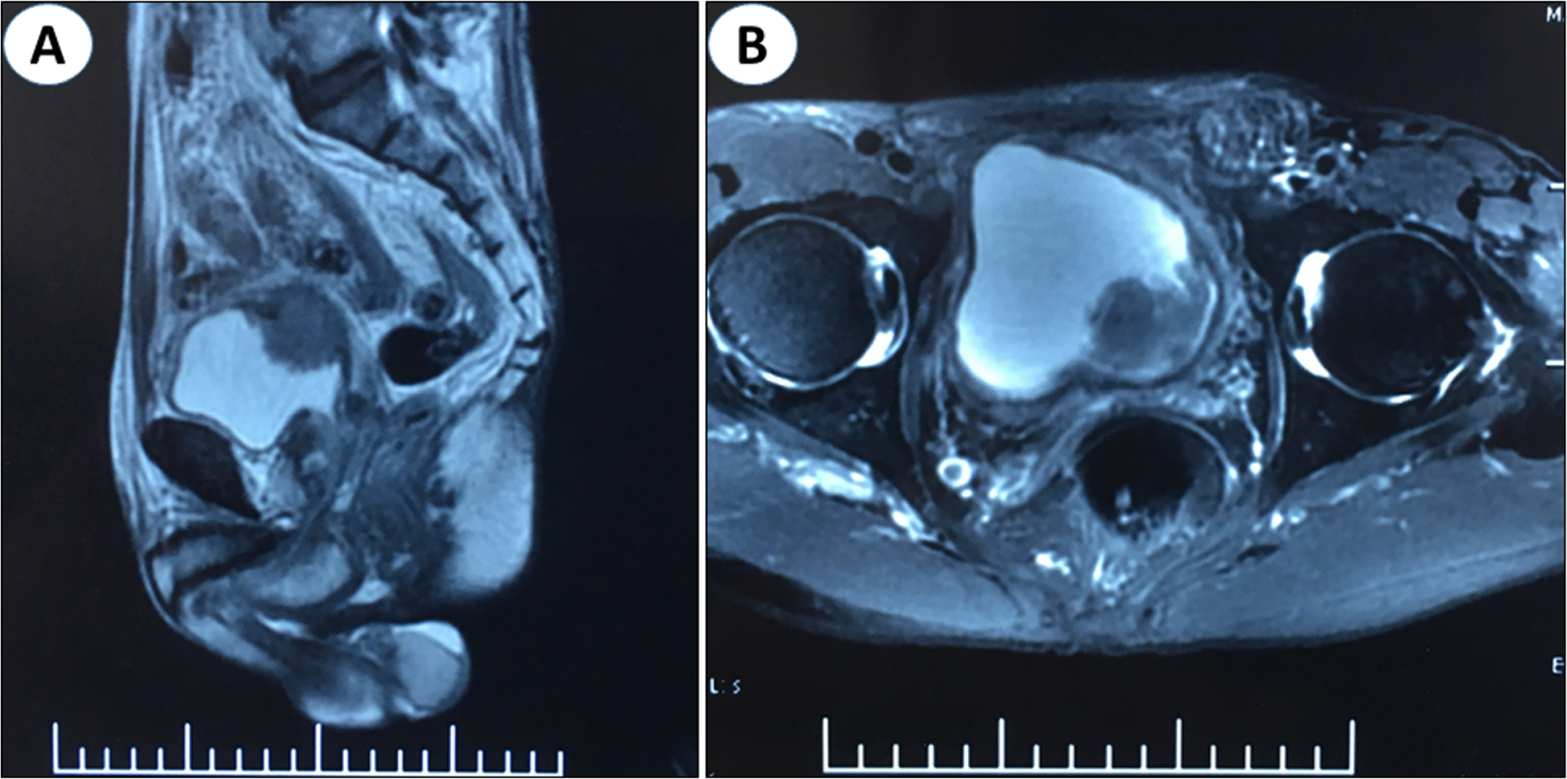
Sagittal (**a**) and horizontal plane (**b**) MRI demonstrated an oval-shaped tumor mass invading the entire layer of bladder in the left posterior bladder wall

A 72-years-old hypertensive man visited our hospital with gross hematuria and odynuria for 2 months (Table 1). Urinary Doppler ultrasound revealed a neoplasm located in the left posterior bladder wall, no enlarged prostate or obvious hydronephrosis. The serum total prostate specific antigen (PSA) was 16.40 ng/ml. The patient stated a smoking history of 40 years and quitted smoking for 8 years. Magnetic resonance imaging (MRI) indicated an oval-shaped tumor mass involving the entire layer of bladder (Fig. 1). Small cell carcinoma and urothelial carcinoma cells were detected through cytological examination.

**Table 1:** The clinical characteristics of two patients

	Case 1	Case 2
Age (years)	72	58
Bladder size (cm)	11.0 × 8.0 × 6.0	10.0 × 6.5 × 5.0
Prostate size (cm)	4.0 × 3.5 × 2.5	5.5 × 4.0 × 3.5
SCCB size (cm)	4.0 × 3.0 × 2.5	3.0 × 2.5 × 1.5 1.5 × 1.0 × 1.0
SCCB location	left posterior wall	posterior wall top wall
Initial symptoms	gross hematuria odynuria	gross hematuria dysuria
Serum PSA (ng/ml)	16.40	8.56

**Fig. 2:**
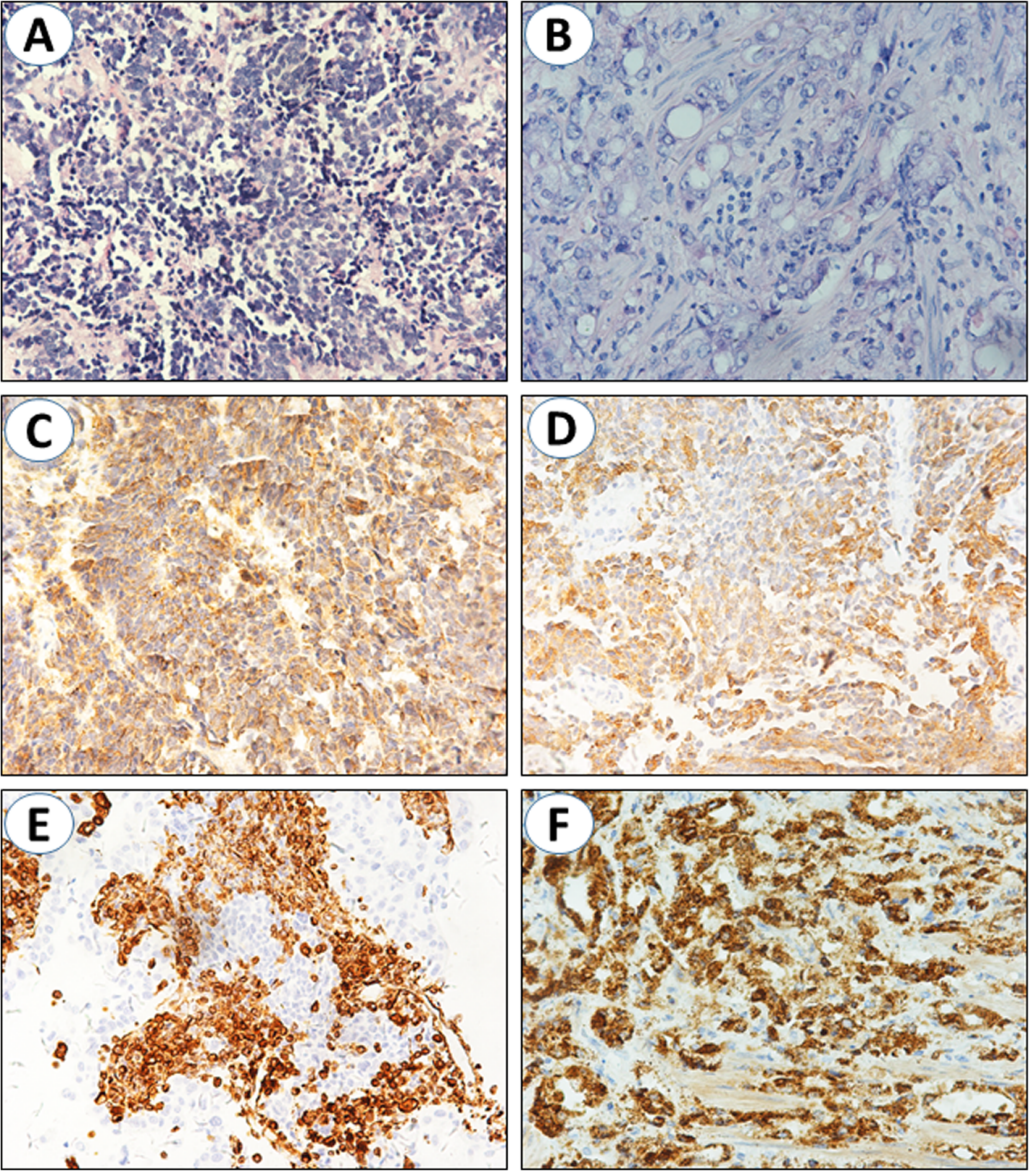
100X magnification of the resected tissues. HE staining showed typical small cells with scant cytoplasm arranged in sheets of SCCB (**a**) and disorder of glands structure, glandular hyperplasia and cell atypia of prostate adenocarcinoma (**b**). Positive staining for CgA (**c**), Syn (**d**), NSE (**e**) and P504s (**f**)

The patient underwent radical cystectomy and prostatectomy. Post-operative pathological examination revealed small round cells containing scant cytoplasm full of the microscope views (Fig. 2a). Mitotis images could be observed as well. Immunohistochemical (IHC) staining displayed intense positivity of neuroendocrine markers, including chromogranin A (CgA), synaptophysin (Syn) and neuron specific enolase (NSE) (Fig. 2c- e). The representative feature of prostate adenocarcinoma was also be discovered, and PSA and P504s were positive (Fig. 2b and f). The IHC results of two cases were shown in Table 2.

**Table 2:** The immunohistochemical (IHC) characteristics of two patients

	Patient 1	Patient 2
IHC staining		
CgA	+	+
Syn	+	+
NSE	+	+
CK7	+	–
LCA	–	–
CD20	–	no
CD3	no	–
P504s	+	+
PSA	+	+
P63	–	–
CK34βE12	–	–
GATA3	+	–
ERG	–	–
TTF-1	–	+
Gleason score	3 + 4 = 7	3 + 3 = 6

The patient accepted regular CE-based chemotherapeutic regimen (Carboplatin, 200 mg, 1d/21d; VP-16, 100 mg, 1-4d/21d) for six sessions and died of liver and lung metastasis 8 months after surgery. The post-operative serum PSA after one and 2 months were 0.34 ng/ml and 0.55 ng/ml, which reminded the biochemical recurrence (BCR) of prostate cancer. During the follow up, the patient accepted endocrine therapy (Bicalutamide, 250 mg/d) as well.

### Case 2 #

**Fig. 3:**
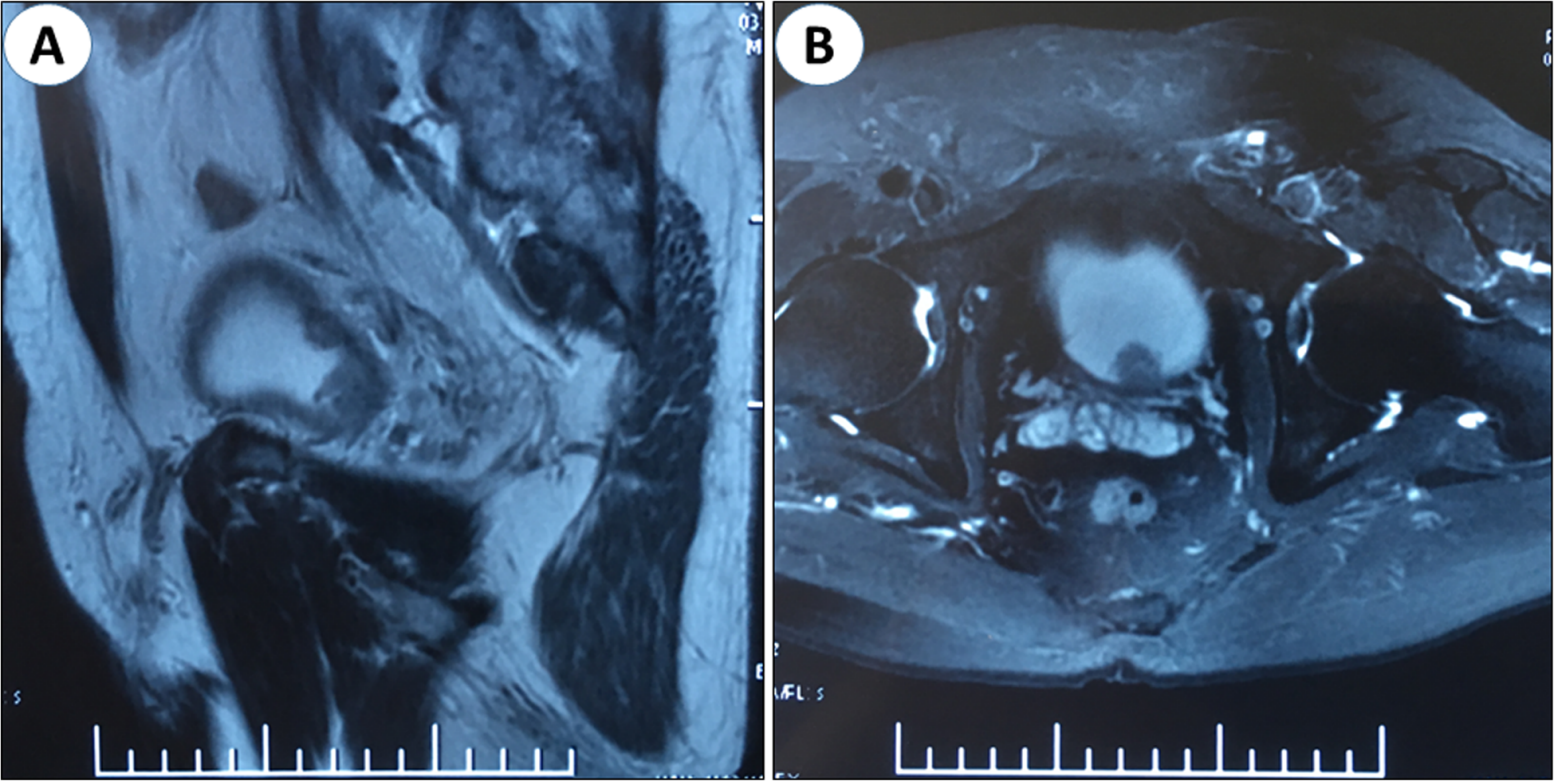
Sagittal (**a**) and horizontal plane (**b**) MRI demonstrated two tumor masses located at the posterior and top wall of the bladder, respectively

A 58-year-old male was hospitalized in our center due to painlessness gross hematuria lasted for 2 weeks and dysuria for half a year. Urinary Doppler ultrasound showed two neoplasms attached to bladder wall, and the prostate volume slightly increased. The serum total PSA was 8.56 ng/ml. MRI subsequently revealed two masses located at the posterior and top wall of the bladder, respectively (Fig. 3). Cystoscopy detection indicated the tumors invaded into the muscle layer. Therefore, we performed radical cystectomy and prostatectomy.

**Fig. 4:**
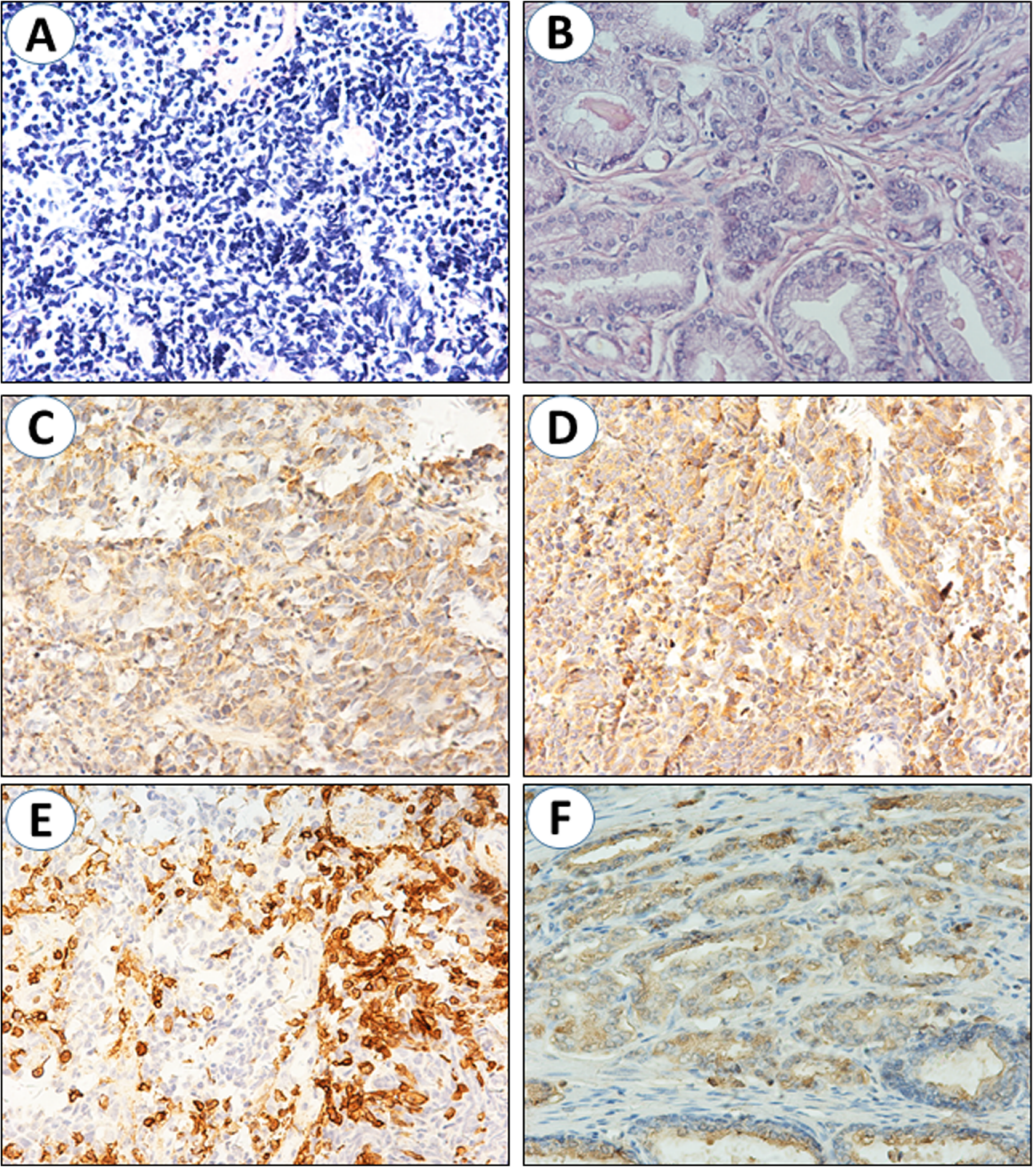
100X magnification of the resected tissues. HE staining showed typical small cells with scant cytoplasm arranged in sheets of SCCB (**a**) and disorder of glands structure, glandular hyperplasia and cell atypia of prostate adenocarcinoma (**b**). Positive staining for CgA (**c**), Syn (**d**), NSE (**e**) and P504s (**f**)

As shown in Fig. 4, pathological examination revealed coexistence of SCCB and prostate adenocarcinoma. CgA, Syn, NSE and P504s were all positive by IHC staining (Fig. 4c-f), while CK7, LCA, CD3, P63 and CK34βE12 were negative (Table 2). The patient received serum PSA examination monthly within 3 months after operation, and the levels were consistently < 0.2 ng/ml. Regular CE regimen was performed for eight sessions. Six months after operation, the patient accepted chest and abdomen CT scan, whichreminded no apparent metastatic lesions in distant organs. The patient has been followed up for 19 months and is still alive up to now.

## Discussion and conclusion

In present study, we described two unusual SCCB cases with combination of prostate adenocarcinoma. HE and IHC staining of tissue sections with demonstration of NSE, CgA, Syn, P504s and PSA supported the diagnosis. Prostate cancer is the most common malignancy in men and a major cause of cancer deaths in western countries [7]. SCCB is extremely infrequent with a proportion about 0.35–0.7% of all bladder tumors, and has a tendency to present with mixed histological accompaniments, like squamous cells, transitional cells and urothelial carcinoma [3, 8]. SCCB has an evident male predominance (male: female = 7: 1) with an average age of 60–80 years [9]. The mechanism of SCCB is still controversial at present. The most widely accepted theory is that it derives from multi-potential stem cells. Such conjecture could also explain the phenomenon that more than half of SCCB coexisted with other histological patterns [10]. The second theory considers that SCCB originates from neuroendocrine cells among the urothelial cells, just like the enterochromaffi cells in the intestinal tract. Another theory suggests it arises from the neuroendocrine cells within the normal or metaplastic urothelium [3, 11].

Bladder tumors often cause hematuria, so unusually subtype of tumors should be kept in mind, especially in elderly patients [12]. Gross hematuria is the most common symptom in SCCB because of the ulcerative growth of invasive lesion into bladder wall. Both the two patients presented with gross hematuria, and odynuria/dysuria. Paraneoplastic syndromes (hypercalcaemia, Cushing syndrome, sensory neuropathy and so on) were commonly in patients of small cell carcinoma of prostate or lung, extremely rare in SCCB [4]. Prostate tumor often cause the elevation of PSA level. According to pre-operative serum chemistries, both patients had higher PSA level than normal (4 ng/ml). Macroscopically, SCCB is usually round or oval without pedicles, and infiltrates into the bladder wall [13]. It is difficult to diagnose SCCB by clinical manifestation or imaging examination. The diagnosis of SCCB mainly depend on histopathological examination. Small and round-shaped cancer cells are representative features of SCCB. Meanwhile, the nest-like structures, overlapping nucleoli, scarce cytoplasm, tumor necrosis and substantial mitotic images are also commonly seen. Neuroendocrine markers are typical for small cell carcinoma, including Syn, NSE and CgA. The appearance of disorder of glands structure, glandular hyperplasia, cell atypia, invasive growth pattern and positive expression of P504S and PSA are essential for the diagnosis of prostate adenocarcinoma [14, 15]. As shown in Figs. 2 and 4, all these pathological characteristics were observed in the resected tissues.

SCCB tends to metastasize to lymph nodes and distant organs early. Koay et al. [16] analyzed 642 SCCB patients and found muscular layer infiltrated in > 90% cases and lymphatic or distant organic metastasis in > 80% cases. But in this study, no obvious metastatic lesions were detected via pre-operative examinations. Proven treatments for SCCB have been explored over the years, while no significant progress has been verified. The curative strategy of SCCB is similar to that of small cell lung cancer (SCLC), with comprehensive treatment including surgery, chemotherapy and radiation therapy. Howbeit, no standard therapeutic regimens have been established currently, and the treatment depends on the stage and progress of SCCB basically [4, 17]. Transurethral resection of bladder tumors (TUR-BT) is not effective enough in controlling SCCB progression, even for localized lesions. Siefker et al. [18] compared the effects of different cooperative therapy (neoadjuvant chemotherapy + surgery vs. surgery + post-operative chemotherapy) for SCCB and found the 5-year survival rates of two groups were 78 and 36%, respectively. Pre-neoadjuvant chemotherapy was able to prolong the survival time of SCCB patients significantly (*p* < 0.05). Carranza et al. [19] also agreed with the benefit of neoadjuvant chemotherapy according to their clinical experience. Pre-neoadjuvant chemotherapy was not used in our study because we could not know the accurate pathological subtype of tumor lesions before operation. Radical cystectomy plus chemotherapy is the most widely used treatment for localized SCCB. At present, cisplatin, cyclophosphamide, VP16 and Adriamycin are frequently used and combined regimen is proven better than monotherapy [20]. Mixed SCCB had a good response to the MVAC protocol (methotrexate + vincristine + adriamycin + cisplatin), while pure SCCB responded to cisplatin-etoposide or ifosfamide/adriamycin regimen better. Since cisplatin has strong side effects of nephrotoxicity and neurotoxicity, it is feasible to choose carboplatin instead of cisplatin in patients with poor conditions [3, 21]. In our study, both patients accepted CE-based chemotherapy regimens after surgery.

Radical prostatectomy (RP) is the optimal strategy for limited prostate cancer. Biochemical recurrence (BCR) is defined as consecutive serum PSA > 0.2 ng/ml twice after RP [22]. Proper treatment for BCR after RP is still controversial, the usual options include radiotherapy of prostate region, anti-androgen drugs in combination with 5α-reductase inhibitors, chemotherapy and observational waiting [22]. In our study, one patient progressed to BCR with successive post-operative serum PSA of 0.34 and 0.55 ng/ml. This patient only took Bicalutamide orally for remedy because of physical and financial reasons, and died of liver and lung metastasis 8 months after operation eventually. Owing to the lack of post-mortem pathological examination, we are not clear whether the metastatic foci derived from prostate adenocarcinoma or SCCB. The other patient just underwent CE chemotherapy regimen after surgery and no BCR of prostate cancer or distant metastasis was detected after a follow up of 19 months.

In conclusion, We reported two extremely unusual cases of coexistence of SCCB and prostate adenocarcinoma and reviewed relative articles in this paper. SCCB is an infrequent pattern of neuroendocrine tumor with low differentiation and high malignancy. There are still many “blind areas” for its pathogenesis, diagnosis and treatment. Although a variety of therapeutic measures are applied currently, the mortality rate of SCCB patients remains high. The discovery of specific diagnostic method and optimal remedial policy should be the keynotes of future research.

## Supplementary information

**Additional file 1.**

## Data Availability

The data and materials of this study are available from the corresponding author on reasonable request. All authors have read the paper and agree that it can be published.

## Abbreviations

SCCB: Small cell carcinoma of the bladder
MRI: Agnetic resonance imaging
PSA: Prostate specific antigen
CgA: Chromogranin A
Syn: Synaptophysin
NSE: Neuron specific enolase
LCA: Leukocyte common antigen
BCR: Biochemical recurrence
IHC: Immunohistochemical
