# Supplementary material for: Small cell carcinoma of the bladder with coexisting prostate adenocarcinoma: two cases report and literature review
Source: BMC Urol. 2020 Aug 28;20:134. doi: 10.1186/s12894-020-00705-3 (PMC7456054; doi:10.1186/s12894-020-00705-3)
Supplement: Supplementary file 1 — Additional file 1. [file 12894_2020_705_MOESM1_ESM.docx]

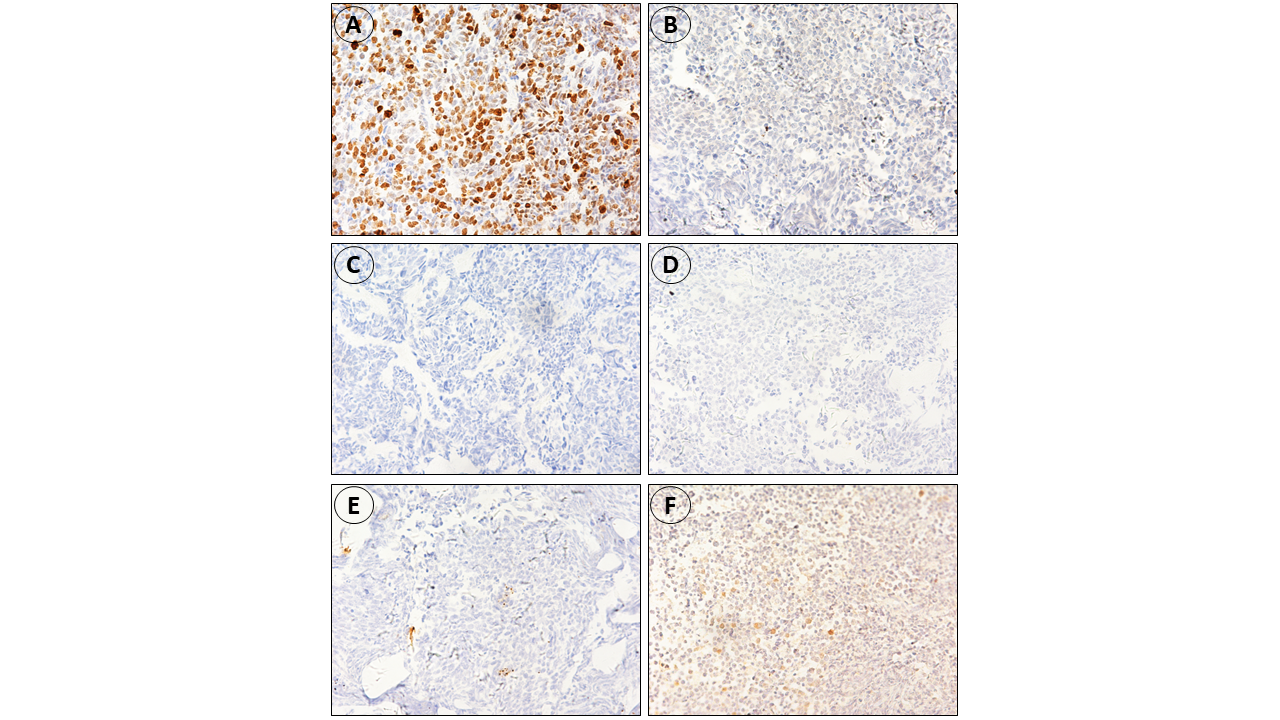


200x magnification of the cystectomy specimens. The GATA3, ERG and TTF-1 expression of case 1# were shown in figure-A, C and E. The GATA3, ERG and TTF-1 expression of case 2# were shown in figure-B, D and F.
